# A Proteomics Approach to Identify New Putative Cardiac Intercalated Disk Proteins

**DOI:** 10.1371/journal.pone.0152231

**Published:** 2016-05-05

**Authors:** Siddarth Soni, Antonia J. A. Raaijmakers, Linsey M. Raaijmakers, J. Mirjam A. Damen, Leonie van Stuijvenberg, Marc A. Vos, Albert J. R. Heck, Toon A. B. van Veen, Arjen Scholten

**Affiliations:** 1 Dept of Medical Physiology, Division of Heart & Lungs, University Medical Centre Utrecht, Utrecht, The Netherlands; 2 Biomolecular Mass Spectrometry & Proteomics, Utrecht Institute for Pharmaceutical Sciences and Bijvoet Center for Biomolecular Research, Utrecht University, Utrecht, The Netherlands; 3 Netherlands Proteomics Centre, Utrecht, The Netherlands; Heart Science Centre, Imperial College London, UNITED KINGDOM

## Abstract

**Aims:**

Synchronous beating of the heart is dependent on the efficient functioning of the cardiac intercalated disk (ID). The ID is composed of a complex protein network enabling electrical continuity and chemical communication between individual cardiomyocytes. Recently, several different studies have shed light on increasingly prevalent cardiac diseases involving the ID. Insufficient knowledge of its composition makes it difficult to study these disease mechanisms in more detail and therefore here we aim expand the ID proteome. Here, using a combination of general membrane enrichment, in-depth quantitative proteomics and an intracellular location driven bioinformatics approach, we aim to discover new putative ID proteins in rat ventricular tissue.

**Methods and Results:**

General membrane isolation, enriched amongst others also with ID proteins as based on presence of the established markers connexin-43 and n-cadherin, was performed using centrifugation. By mass spectrometry, we quantitatively evaluated the level of 3455 proteins in the enriched membrane fraction (EMF) and its counterpart, the soluble cytoplasmic fraction. These data were stringently filtered to generate a final set of 97 enriched, putative ID proteins. These included Cx43 and n-cadherin, but also many interesting novel candidates. We selected 4 candidates (Flotillin-2 (FLOT2), Nexilin (NEXN), Popeye-domain-containg-protein 2 (POPDC2) and thioredoxin-related-transmembrane-protein 2 (TMX2)) and confirmed their co-localization with n-cadherin in the ID of human and rat heart cryo-sections, and isolated dog cardiomyocytes.

**Conclusion:**

The presented proteomics dataset of putative new ID proteins is a valuable resource for future research into this important molecular intersection of the heart.

## Introduction

Evidence of the heart being a functional syncytium was first provided in the 19^th^ century and this was later followed by the idea that cardiomyocytes were connected via low cell-to-cell resistances [1]. An important structure involved in providing these low cell-to-cell resistances is the intercalated disk (ID) [2,3]. The ID is a highly coordinated and complex structure with two main functions a) to maintain mechanical and metabolic coupling, and b) to enable fast propagation of electrical impulses throughout the heart. The currently known structural components of the ID are classically divided into three parts: the desmosome, the fascia adherens (FA) and gap junctions (GJ). Recently a fourth region, the transitional junction (TJ), was described at the perimeter of the FA although to date information is scarce about the exact role of this last ID component [4]. Resulting from this last observation, the ID currently is considered as a large and highly orchestrated macromolecular protein complex in which the different entities are physically integrated rather than being distinct.

Cardiac muscle is subjected to high contractile forces, and is therefore rich in desmosomes to provide structural support between myocytes. The desmosomes, are rich in the transmembrane proteins desmocollin and desmoglein which form a dense and robust network to connect the IDs of adjacent cardiomyocytes [5]. On the cytosolic side, the desmosome is composed of desmoplakin and plakoglobin (also known as γ-catenin) which link the desmosome to the cytoskeleton [6].

The FA links the ID to the myofibrils to facilitate uniform mechanical strength to the heart [7], by supporting the transmission of contractile force from one cell to another [8]. At the molecular level, the FA is enriched in the transmembrane protein n-cadherin and members of the catenin family of proteins on the cytoplasmic side [9].

GJs are present in nearly all cell types within the body and in the ID of cardiomyocytes they contribute to chemical and electrical cell-to-cell coupling, and impulse propagation, by connecting the cytoplasm of neighbouring cells. The GJ channels between two cardiomyocytes, all consist of two hemichannels named connexons, which in turn are formed through hexagonal arrangements of connexin proteins [10,11]. Connexin43 is the most abundant connexin isoform in the ventricular myocardium [12]. Because of a low resistance maintained by the GJ compared to the membrane [13], the channels are permeable to ions and signalling molecules up to a size of about 1 kD such as Ca^2+^, cAMP and IP_3_ [14]. By this they facilitate metabolic coupling.

These described functions of the ID suggest it is an important structural component for the synchronised beating of the heart, but therefore also a putatively vulnerable area involved in cardiac disease. Indeed, in many cardiac diseases alterations in composition of the ID are related to development and progression of the phenotype. This not only includes classical diseases like dilated-, or ischemic cardiomyopathy (DCM and ICM, respectively) but also arrhythmogenic right ventricular cardiomyopathy (ARVC). This latter disease nowadays is also mentioned as arrhythmogenic cardiomyopathy (ACM) and is characterized by the fact that a high number of those patients die suddenly due to rhythm disorders [15]. Primarily responsible for this disease are mutations in desmosomal proteins like plakophilin-2 and desmoglein-2 are [16–21]. The incomplete knowledge regarding the molecular composition of the ID is a major bottleneck in understanding the underlying mechanisms of such diseases and as such hampers the development of suitable treatments.

The complex organization of the ID lead us to hypothesize that there are many more proteins yet to be discovered to completely understand the composition and functioning of the ID. To date, around 200 proteins have been reported to reside in the ID based on existing literature (reviewed by Estigoy *et*. *al*. [22]). The presence of these 200 ID proteins primarily has been validated by cardiac immunohistochemistry and these data have been collected within the human protein atlas (www.proteinatlas.org). Although this is a powerful approach, it did not bring forward novel players.

In the current study, using biochemistry, advanced proteomics technology and *in silico* prediction, we have applied an approach with the aim to identify putative novel components of the ID. Based on an in-depth analysis of cardiac membrane fractions, including the ID proteins, we put forward a dedicated, stringently filtered, library of 97 (novel) putative ID proteins, of which we have currently validated four by complementary biochemical and imaging techniques.

## Materials and Methods

### Animals and animal procedures

Animal care and handling was performed in accordance with the ‘European Directive for the protection of Vertebrate animals used for Experimental and Scientific Purpose, European Community Directive 86/609/CEE’. All experiments were approved by the committee for experiments on Animals of the Utrecht University, The Netherlands. Male Wistar rats were housed at 21°C and 60% humidity with an artificial 12:12 h light dark cycle and were fed with standard chow and water (ad libitum). Rats were anaesthetized with 2.5% isoflurane in 40% oxygen and heparin (1 ml, 5000 I.U, IP) was administered to avoid the formation of any coagulation. Hearts were extracorporated by creating an incision in the thoracic cavity. Subsequently, the aorta was canulated and hearts were flushed with PBS to remove any remaining blood. Finally, hearts were rapidly frozen in liquid nitrogen and immediately stored at -80°C until further use.

### Human tissue

From the biobank of the Department of Pathology, University Medical Center Utrecht, left ventricular material tissue was obtained from healthy control male individuals that died without any diagnosed cardiovascular disorders (explanted hearts not used for transplantation due to logistical reasons), and from male patients that were diagnosed with dilated cardiomyopathy (DCM) or Arrhythmogenic right ventricular cardiomyopathy (ARVC). Tissue was obtained from explanted hearts at the moment of cardiac transplantation and directly frozen in liquid nitrogen after extra-corporation. All investigations conform to the principles outlined in the Declaration of Helsinki and experimental protocols were approved by the Ethical Review Boards of the University Medical Center Utrecht (The Netherlands).

### Isolation of enriched membrane fractions

Frozen rat hearts (n = 3) were thawed for 10 minutes at 4°C followed by careful dissection of the left ventricle (LV). The LV was cut into smaller pieces and homogenized in lysis buffer (20mM tris HCL pH 7.4, 1mM EGTA, 50mM NaCl, 5mM NaN_3_, 1mM PMSF, 50mM Na_3_VO_4_ and a protease inhibitor cocktail (Roche, Germany) using a glass Teflon homogenizer. The lysate was centrifuged at 500g to remove all unbroken cells and nuclei, supernatant was collected and again centrifuged at 10,000g. The resulting pellet was collected as the enriched membrane fraction (EMF) and dissolved in lysis buffer (constituted as mentioned above). The supernatant being the cytosolic fraction (CF) was used as a control to estimate enrichment levels of proteins in the EMF and hence, for the following proteomics analysis two fractions were used; the EMF and CF. A similar approach was used for isolation of human EMFs from LV free wall tissue (LVFW).

### Western blotting

Total protein was isolated from ventricular tissue as described previously [23]. Briefly, 20μg of protein lysate was separated by a 10% sodium dodecyl sulfate-polyacrylamide gel electrophoresis (SDS-PAGE). Resolved proteins were electro-transferred onto nitrocellulose membranes (0.45μM, Bio-Rad Laboratories). Equal protein loading was verified by Ponceau S staining. Membranes were incubated overnight with primary antibody (Cx43 1:250, BD Bioscience 610062; Flotillin-2, 1:250, Thermo Scientific PAS-21296; POPDC2, 1:1000, Sigma HPA024255, GAPDH, Millipore, MAB374), followed by incubation with the appropriate secondary antibody. Proteins were visualized using a standard ECL procedure (GE Healthcare).

### LC-MS/MS analyses

Protein samples (250μg per heart) were reduced and denatured by heat at 96°C for 10 minutes in 150 μl of 1X sample buffer (Invitrogen). The proteins were separated on a 4–15% gradient gel (Bio-Rad, Germany) and Coomassie stained. Each equivalently loaded gel lane (3x EMF, 3x CF) was cut into 15 equal bands, which were subsequently reduced, alkylated and in-gel digested as described previously [24].

Peptides were separated on an in-house made 50 cm column, 75 μm inner diameter, packed with 1.8 μm C18 resin (Agilent Zorbax SB-C18) at a constant temperature of 40°C. The column was connected to a Q Exactive quadrupole orbitrap mass spectrometer (Thermo Scientific) through a nanoelectrospray ion source.

Injected peptides were first trapped with a double fritted trapping column (Dr Maisch Reprosil C18, 3 μm, 2 cm x 100 μm) at a pressure of 800 bar with 100% solvent A (0.1% formic acid (FA) in water) before being chromatographically separated by a linear gradient of buffer B (0.1% FA in acetonitrile) from 7% up to 30% in 150 min at a flow rate of 150 nl/min. Total measurement time for each sample took 180 minutes. The eluent was sprayed via a distal coated fused silica emitter (360 μm o.d., 20 μm i.d., 10 μm tip i.d.; constructed in-house) butt-connected to the analytical column.

Mass spectra were acquired on the Q-Exactive in data dependent mode with an automatic switch between a full scan and up to 20 MS/MS scans. Target value for the full scan MS spectra was 3,000,000 with a maximum injection time of 250 ms and a resolution of 35,000 at m/z 200. The twenty most intense ions with charge two or more from the survey scan were selected with an isolation window of 1.5 m/z and fragmented by HCD with normalized collision energies of 25. The ion target value for MS/MS was set to 50,000 with a maximum injection time of 120 ms and a resolution of 17,500 at m/z 200. Repeat sequencing of peptides was kept to a minimum by dynamic exclusion of the sequenced peptides for 40 s.

### Data analysis

For the label free quantitative analysis, the raw files were processed using MaxQuant version 1.3.0.5 [25]. The MS/MS spectra were searched against the IPI rat database 3.36 (42,689 sequences; 22,370,705 residues) using the Andromeda search engine.

Database search was performed with the following parameters: an initial mass tolerance of ±20 ppm for precursor masses and a final mass tolerance of ±7 ppm; a mass tolerance of ±0.05 Da for HCD fragment ions, and up to two missed cleavages were allowed. Cysteine carbamidomethylation was used as a fixed modification and methionine oxidation and protein N-terminal acetylation as variable modifications. For the identification, the false discovery rate was set to 0.01 for peptides and proteins. The minimum peptide length allowed was set to seven amino acids. The match between runs feature was switched on. The mass spectrometry proteomics data have been deposited to the ProteomeXchange Consortium via the PRIDE partner repository [26], with the dataset identifier PXD000947.

To isolate putative ID proteins from the proteins identified in the EMF we carried out a bioinformatics approach. First, we calculated an enrichment ratio (ER) using the average intensities of the label free quantitation (LFQ) of the three EMFs divided by the average of the LFQ intensities of the three CF (ER = Avg. ID LFQ/Avg. CF LFQ intensity). Using the ER values we generated a prioritized protein list. These were selected based on the following criteria: i) an ER>10, ii) identified in all 3 EMF replicates and, iii) at least identified with 5 peptide spectrum matches in one of the 3 replicates. A particular set consisted of, proteins that were uniquely present in all three EMFs, while completely absent in all three CF fractions. These were given an arbitrary ER = 100. These EMF proteins were analyzed further using the Biogrid database for protein-protein interactions using data from either human, mouse and rat (version 11/11/13). Subcellular localizations and Gene Ontology terms (GOSlim) for each protein were obtained from the BioMart R package from Bioconductor. Network visualization was done using Cytoscape and the Cytoscape plugin Cerebral for clustering based on cellular localization.

### Immunohistology

Left ventricle sections (10μm) were obtained from human healthy individuals (without any diagnosed cardiovascular disease) and control rats [27]. Isolated cardiomyocytes were from dogs as described in [28]. Immunolabeling was slightly modified according to Andrée et al. [29]. Sections or cells were fixed in 2% paraformaldehyde for 10 min. and subsequently washed 3 times for 5 min. in PBS. Subsequently, samples were permeabilized using 0.25% Triton X100 in PBS for 5 min. and blocked in blocking solution (1% BSA, 5% normal goat serum, 0.25% Triton X100 in PBS) for 1h at RT. Next, the samples were incubated with primary antibodies (Flotillin-2, 1:100, Thermo Scientific PAS-21296; Nexilin, 1:50, Abcam AB83746; POPDC2, 1:50, Sigma HPA024255; TMX2, 1:50, Sigma HPA040282; N-Cadherin, 1:800, Sigma C3678). Samples were washed 3 times 5 min. in PBS prior to incubation with the appropriate secondary antibody in blocking solution for 1h at RT. Sections were analyzed on a widefield Nikon microscope (Eclipse 80i). Images were captured with a Nikon camera (DS-Fi1) at 400x magnification, using NIS Elements BR3.0 software, and deconvoluted using Huygens Essential 4.1 software (Scientific Volume Image B.V.) For quantification of co-localization, the two fluorescence profiles were compared with ImageJ 1.43 by plotting a line through the visible signals and create two plot profiles. These were compared, and a correlation coefficient (R^2^) was computed using MS Excel.

## Results

**Fig 1:**
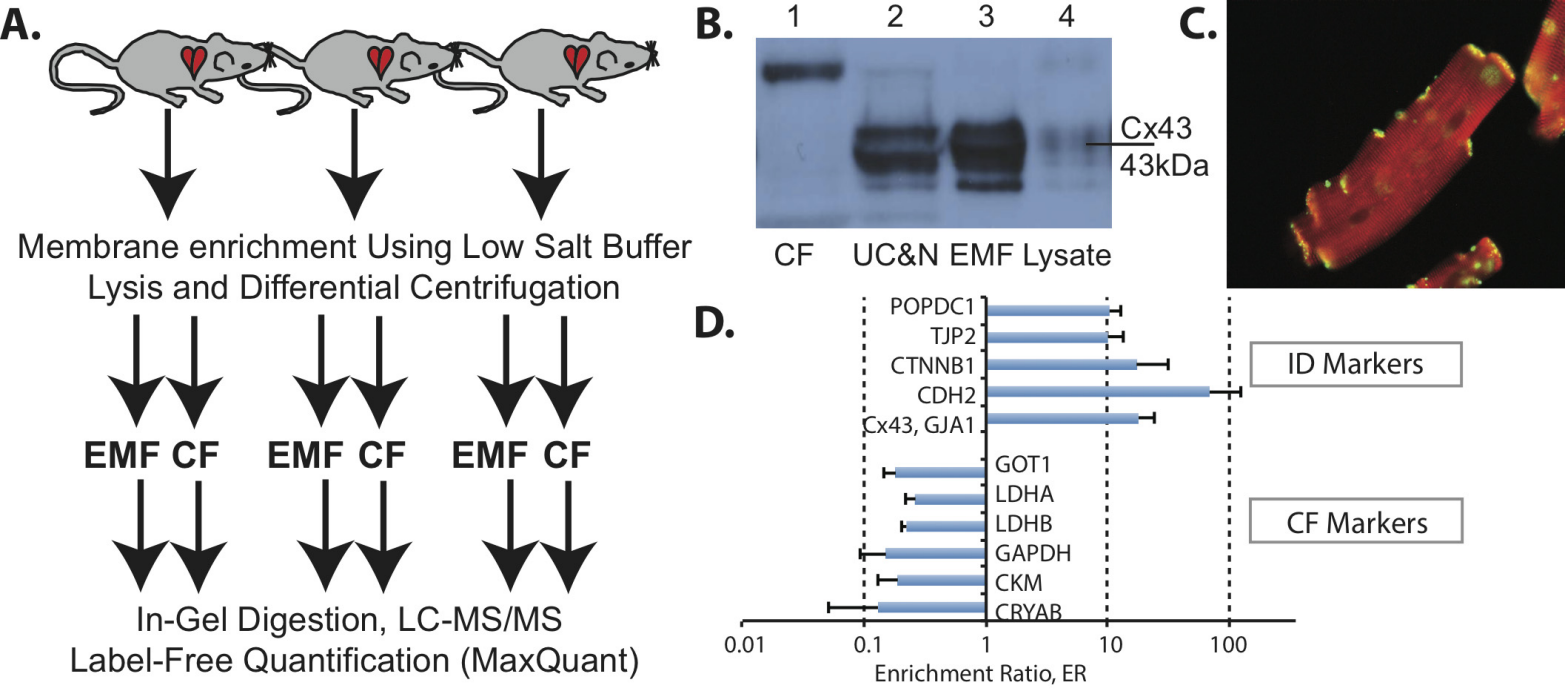
Strategy to identify putative intercalated disk proteins. (A) Schematic representation of the approach followed to identify intercalated disk proteins in the enriched membrane fraction (EMF). The cytosolic fraction (CF) was used as a control to quantify enrichment levels. Enrichment was done using differential centrifugation steps. Samples were digested in-gel and subsequently analyzed by LC-MS/MS and label free quantitation using MaxQuant software [25]. (B) The EMF is compared to different fractions obtained during the enrichment process as visualized by connexin-43 (Cx43) Western blot. (CF = cytosolic fraction, UC&N = unbroken cells and nuclei, EMF = enhanced membrane fraction, lysate = entire cardiac tissue lysate) (C) Immunostaining of Cx43 (green) on cardiomycytes, with alpha actin staining used as reference (red). (D) Enrichment ratios (ER) of selected CF and ID markers and the log value of their standard deviations (n = 3). Depicted are Alpha-crystallin B chain (CRYAB), Creatine Kinase M (CKM), Glyceraldehyde-3-phosphate dehydrogenase (GAPDH), L-lactate dehydrogenase B chain (LDHB), L-lactate dehydrogenase A chain (LDHA), Aspartate aminotransferase, cytoplasmic (GOT1) as CF markers and Connexin-43 (Cx43, GJA1), N-Cadherin (CDH2), Beta-Catenin (CTNNB1), Tight junction protein 2 (TJP2) and Blood vessel epicardial substance (POPDC1) as known ID markers within the EMF.

Currently, it is not possible to specifically enrich the ID membrane fraction from cardiac tissue lysates in a consistent manner. Therefore, in this study we designed an alternative strategy by preparing enriched cardiac membrane fractions (EMFs) from rat ventricular tissue in triplicate and analyzed these in-depth by mass spectrometry based proteomics. The EMFs were compared to corresponding cytosolic fractions (CFs) in a quantitative fashion using ion intensity based information from the proteomics experiments as described previously [30, 31] (Fig 1A). Subsequently, an intracellular location driven bioinformatics approach was amended to further filter putative novel ID proteins from the general membrane proteins present in the EMF.

### Cardiac membrane fraction characterization

Prior to proteomic analysis, the cardiac EMFs were tested for presence and enrichment of the known ID marker Cx43. Cx43 was clearly enriched in the EMF. Cx43 was not, or at a lower level, present in the cytosolic and whole lysate samples respectively (Fig 1B, S3 Fig-I). Fig 1C shows Cx43 immunostaining in a cardiomyocyte at the ID and the alpha actin staining (cross-striations) displays the cellular architecture aiding to identification of the ID.

### Proteomic analysis of the EMF and CF

**Fig 2:**
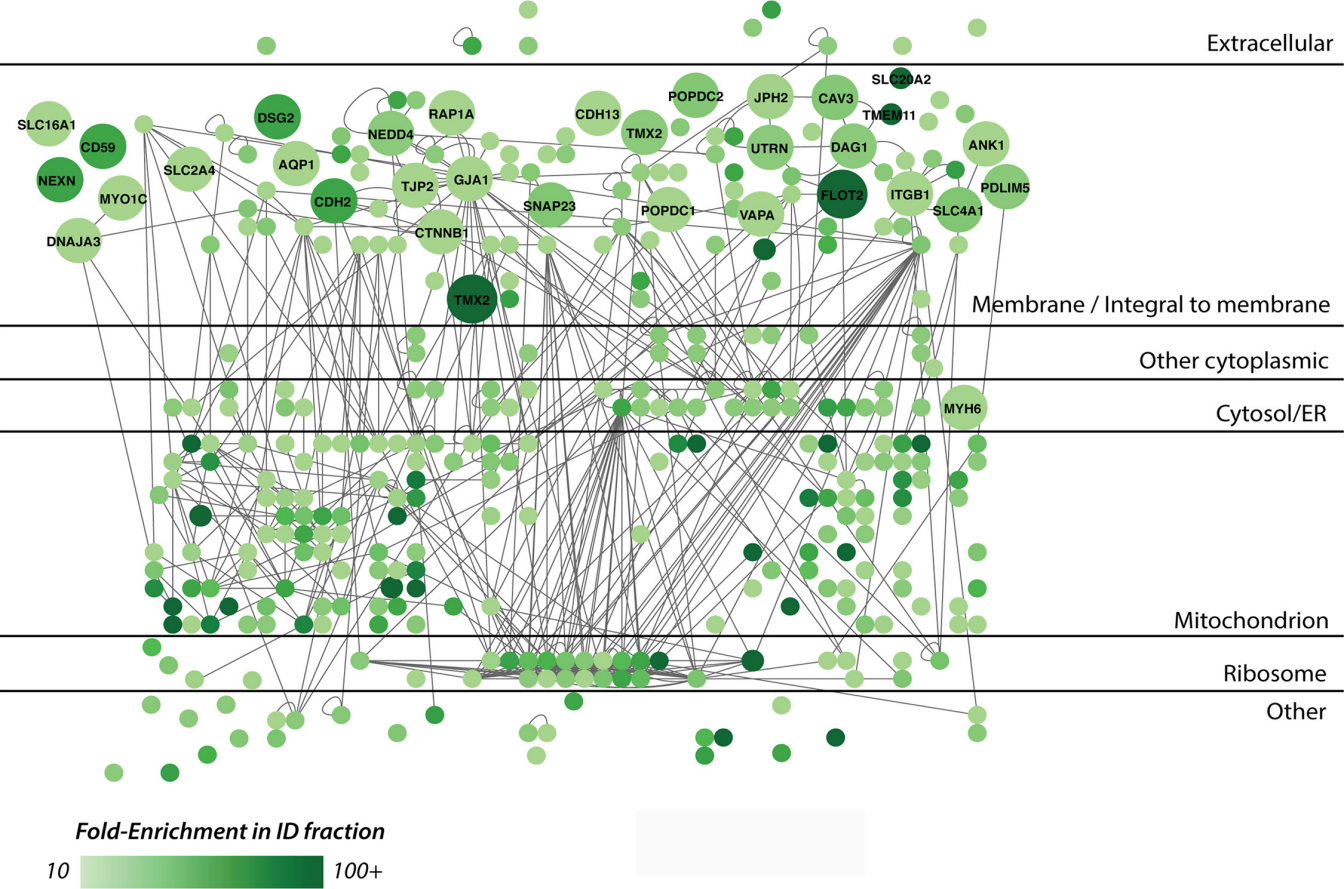
Molecular context of proteins in the EMF. Schematic representation of protein-protein interactions and cellular localization across the 366 candidate proteins that showed enrichment (ER>10-fold) in the EMF-fraction. This highly connected network is based on protein-protein interactions currently available in the Biogrid database in mouse, rat and human (lines). The color of the nodes represents the fold-enrichment measured; darker colors mean higher enrichment ratios. All known cell junction proteins, as based on GO-terms and a recent *in silico* study by Estigoy et al. [22], are visualized by their gene names and enlarged circles. As expected, these mainly clustered in the plasma membrane region and several known ID protein complexes were readily identified at this intracellular location. These and all other (connected) proteins in this compartment were considered as most likely ID candidates and further evaluated in Fig 3.

**Fig 3:**
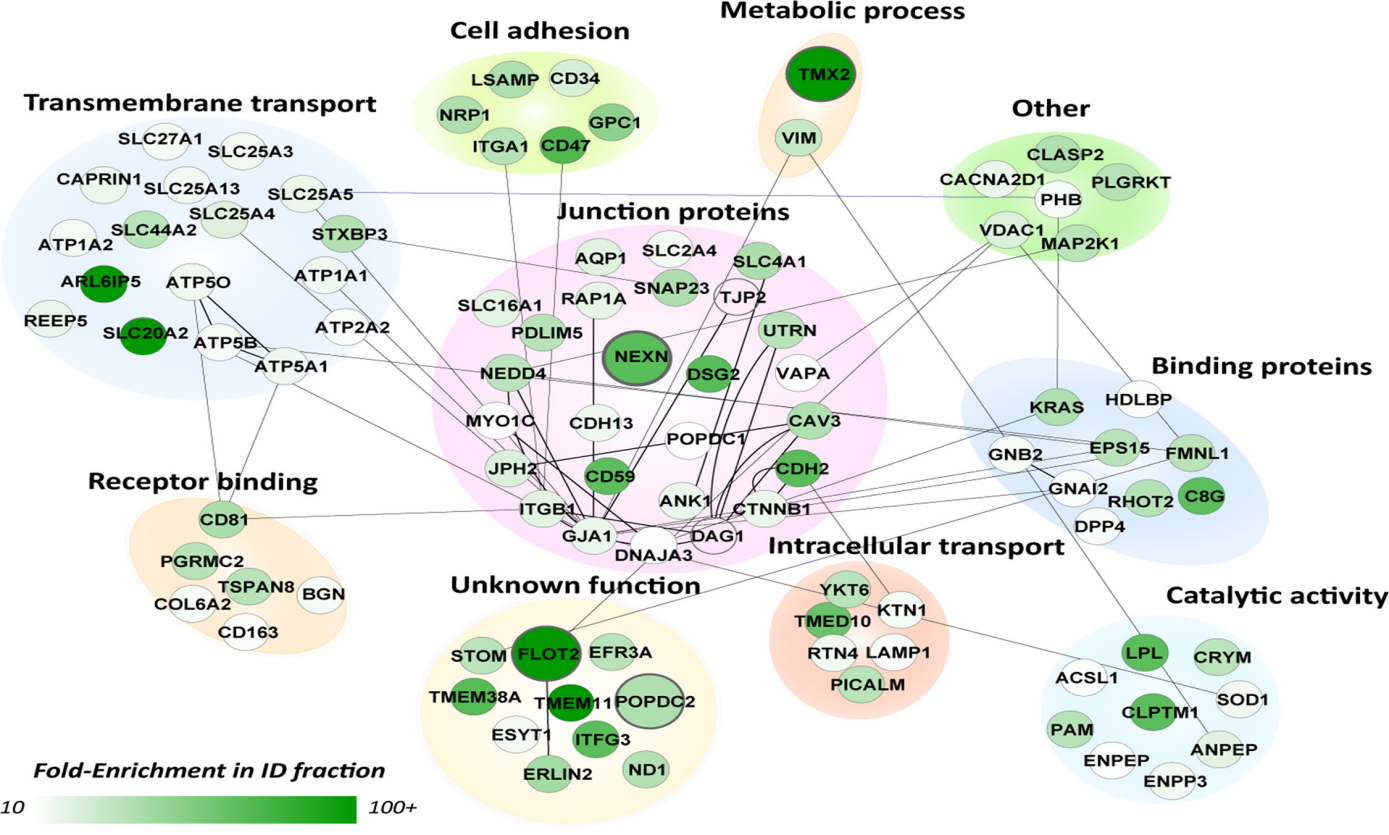
Molecular functions of designated ID proteins. Subnetwork GO analysis of the 97 candidate proteins observed in the membrane or integral to membrane compartment in Fig 2. Proteins were clustered according to their GO annotation in process and function. Comparable to Fig 2, the color of the nodes represents their fold-enrichment measured (ER). The cluster in the middle contains all currently known junction proteins (based on GO and Estigoy et al. [22]). Proteins in this cluster are connected to proteins with other biological functions based on known protein-protein interactions (lines). The four candidate proteins selected for follow-up studies are enlarged.

Proteomic analysis of the EMFs and CFs revealed a total of 3455 proteins (S1A Table), of which 1882 were present both in the CF and the EMF. Six-hundred-forty-seven proteins were found uniquely in the EMF and 926 were unique in the CF. In order to extract putative ID proteins we utilized an intuitive data-filtering scheme based on stringent criteria. These were based on the quotient of the label-free quantitation derived intensities in the EMFs and CFs (enrichment ratio (ER)) and spectral count criteria following established workflows [30, 31]. The final criteria consisted of: 1) ER≥10, 2) each candidate identified in all three EMF fractions and 3), at least one EMF identification with a minimum of 5 peptide spectrum matches (see materials and methods for details). In this way we could identify 366 candidate proteins to be specifically enriched in the EMF with high confidence (S1B Table). Besides Cx43, this fraction contained many more established ID markers with average ER values above our stringent ER cutoff of 10-fold (Fig 1D). Typical cytosolic proteins consistently showed opposite ER values. As expected from our general membrane enrichment approach, several different membrane rich areas were purified from the ventricular tissues, including mitochondrial, golgi and endoplasmic reticulum components. Therefore, the 366 initial candidates were further evaluated for their intracellular localization and their documented protein-protein interactions (Fig 2). This resulted in a highly connected network as based on protein-protein interactions currently available in mouse, rat and human. Subsequently, all known cell junction proteins, as based on GO-terms and a recent *in silico* study by Estigoy et al. [22], were visualized. As expected, these mainly clustered in the plasma membrane region and several known ID protein complexes were readily identified at this intracellular location, such as Cx43 (GJA1) and the N-Cadherin (CDH2)–Beta-Catenin (CTNNB1) complexes, consistent with previous reports [27,32–34]. Other, known ID markers included, ankyrin-1 (ANK1) and desmoglein-2 (DSG2) [12]. The proteins termed membrane/integral to membrane were isolated (97 proteins, S1C Table) and studied further using their documented functions (Fig 3). Most proteins clustered in two main categories, junction proteins (26 proteins) and transmembrane transport (17 proteins). The former contains many known markers such as Cx43 (GJA1), N-Cadherin (CDH2), beta-catenin (CTTNB1), but also tight junction protein 2 (TJP2) and blood vessel epicardial substance (POPDC1, synonym of BVES1). Other categories included cell adhesion (6 proteins), intracellular transport (6 proteins), catalytic activity (9 proteins), metabolic processes (2 proteins) and receptor binding (6 proteins). We also found 10 proteins with a currently unknown function. From these 97 candidate ID proteins we selected 4 candidates based on a high ER and a currently unknown function in, or an undocumented connection to the ID: Flotillin-2 (flot2, ER = 100-fold), Popeye domain containing 2 (POPDC2, ER = 38-fold), Nexilin (NEXN, ER = 68-fold) and Thioredoxin-related transmembrane protein 2 (TMX2, ER = 100).

### All four selected candidate proteins localize to the cardiac ID

**Fig 4:**
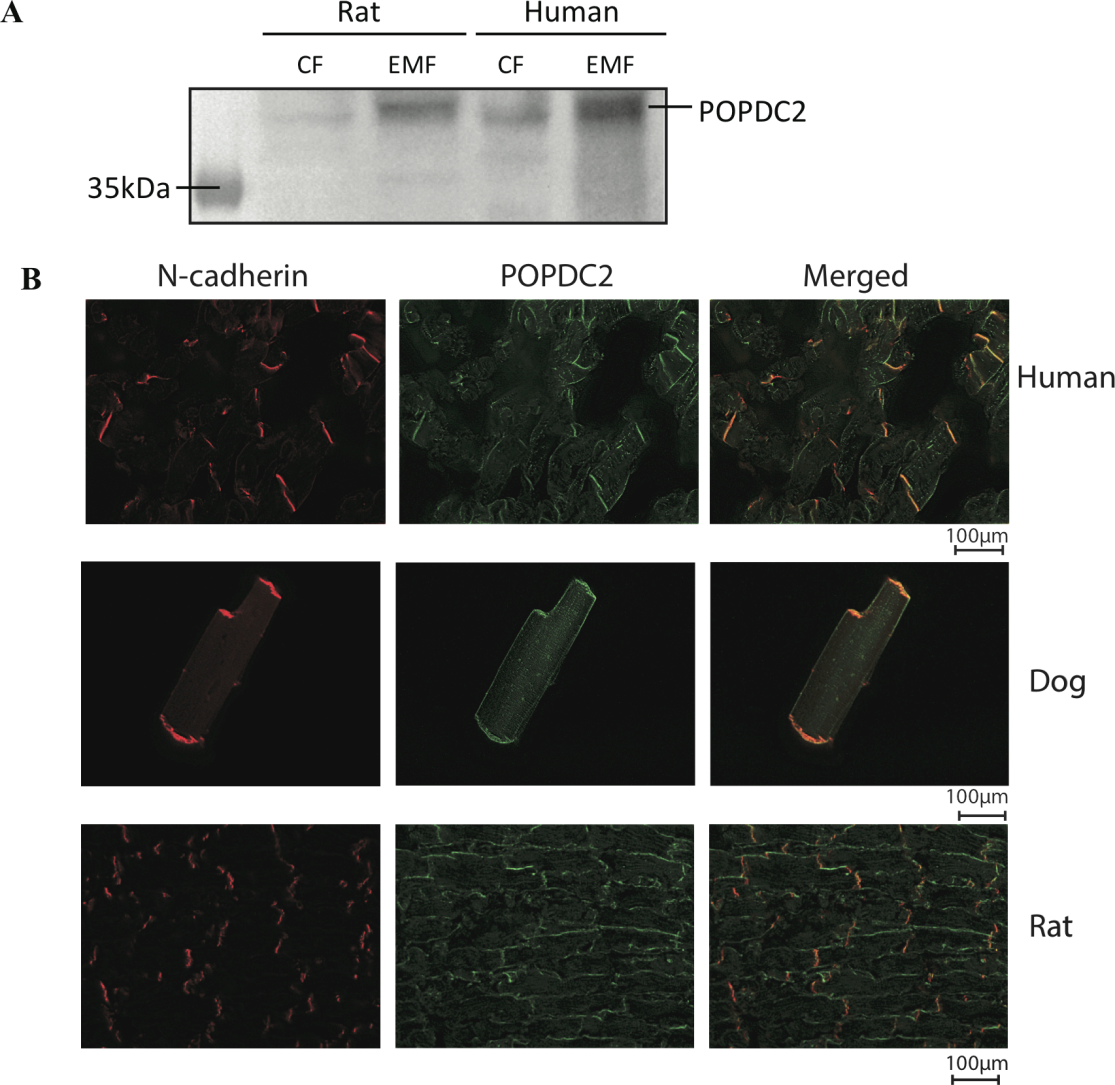
Popdc2 localizes to the intercalated disk. (A) Western blot showing that POPDC2 is enriched in the EMF compared to the CF in both rat and human left ventricular tissue. (B) Immunofluorescence imaging of N-cadherin and POPDC2. Merged images (overlay, right) show clear co-localization of N-cadherin and POPDC2 in human left ventricular tissue, isolated dog cardiomyocytes and rat left ventricular tissue at the ID.

**Fig 5:**
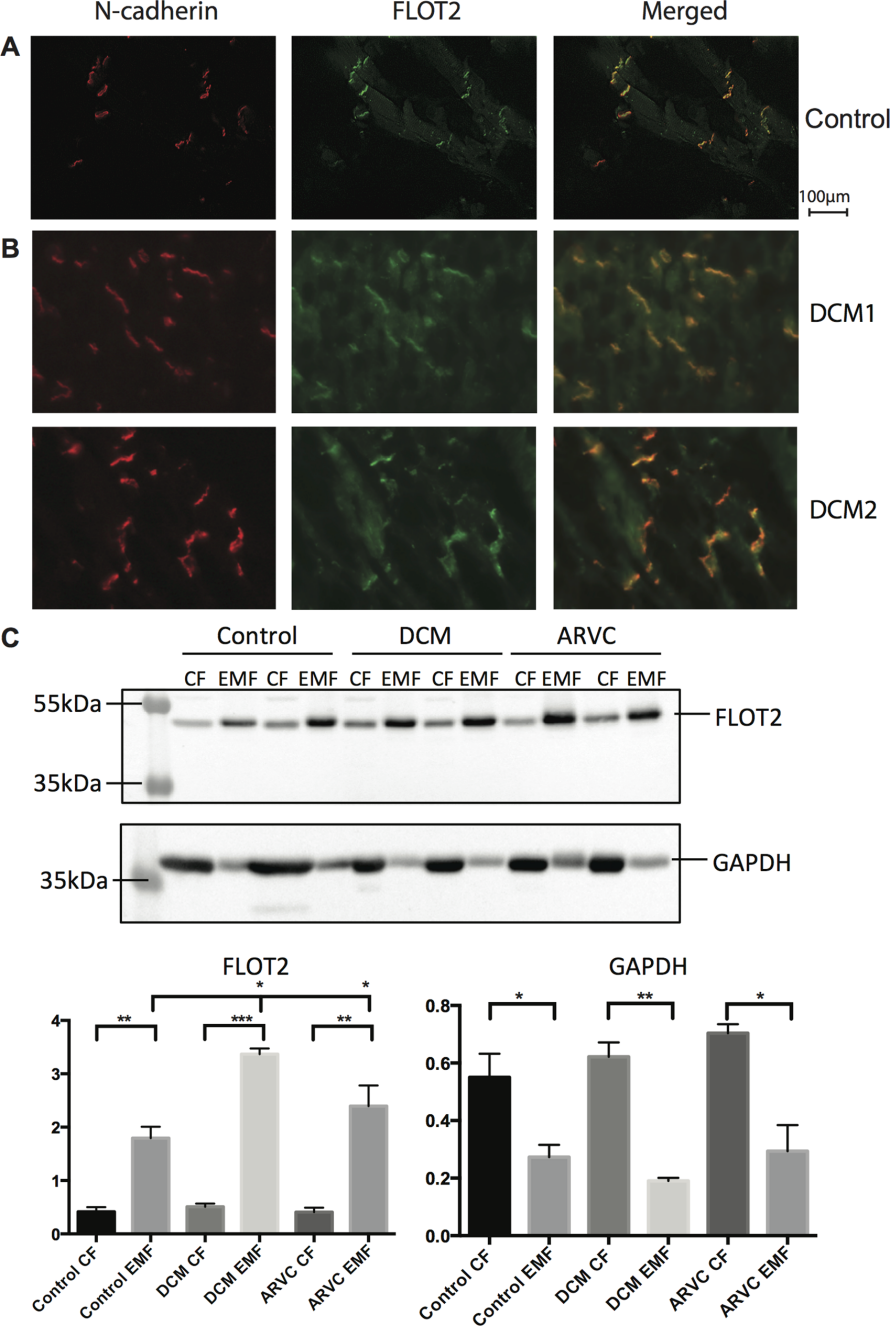
Flotillin-2 shows increased ID expression during cardiac disease. (A) Immunofluorescence imaging of N-cadherin and flotillin-2 in human left ventricular tissue. The overlay image reveals strong co-localization. (B) Co-localization studies of N-cadherin and flotillin-2 in left ventricular tissue from two patients with DCM hints at an increased level of flotillin-2 at the ID. Intensity and localization of fluorescent signals were analyzed through line scans and are plotted for both patients (S2 Fig). Robust co-localization was confirmed through correlation coefficients of R^2^ = 0.81 and 0.86 for the upper panel and lower panel, respectively. (C) Representative Western blots of the CF and EMF in healthy human tissue, compared to left ventricular tissue of patients with either DCM or ARVC. Differences between the levels in CF and EMF were analyzed using an unpaired t-test. Differences between the groups of patients were analyzed via a one-way ANOVA. * P<0.05, ** P<0.005, ***P<0.0005.

Immunohistochemistry was performed to confirm the presence of the four candidate proteins at the ID in three different species; human, rat and dog. All four candidates: POPDC2 (Fig 4B), TMX2, (S1A Fig), NEXN (S1B Fig) and FLOT2 (Fig 5A, S1C Fig) showed clear signals at the ID and a strong co-localization with N-Cadherin in all species. Labeling against POPDC2 revealed some additional lateral signals, while the antibody used against NEXN did not reveal any immunolabeling in human tissue.

### Increased ID expression of Flotillin-2 at the ID under diseased conditions

To start gaining insight into the potential importance of flotillin-2 at the ID we investigated its presence at the ID under both healthy and diseased conditions. Therefore we investigated the localization of FLOT2 further in left ventricular tissue samples obtained from two dilated cardiomyopathy patients (DCM) (Fig 5B). Immunolabeling and the quantification profiles (S2 Fig) hinted at an increased level of FLOT2 in diseased IDs. To substantiate this finding, the enrichment protocol was repeated on left ventricular tissues of healthy controls, DCM patients and patients with arrhythmogenic right ventricular cardiomyopathy (ARVC) and the protein levels were profiled by Western blot (Fig 5C, S3 Fig-III). Quantification of these signals confirmed a significantly higher level of FLOT2 in the EMF for all three groups. Moreover, the intensity in the EMF of DCM and ARVC was significantly higher as compared to control whereas the much lower intensities of FLOT2 in the CFs were similar. Inversely, signal intensity of GAPDH was significantly higher in the CFs off all three groups when compared to their respective EMF, whereas no statistical differences between both the EMFs and the CFs in between the three groups could be observed.

## Discussion

With an increased prevalence of cardiac diseases related to dysfunctioning of molecular constituents of the ID, the urge for a more complete knowledge of the ID composition has become vital. The main hurdle is the lack of protocols to understand the composition of the ID as a whole and up till now there have been no comprehensive studies conducted to actually identify novel ID proteins and understand the composition of this entire complex structure [22]. This study is the first of its kind where membrane fractions, with the ID-components included, were isolated and analyzed in-depth by mass spectrometry-based proteomics and bioinformatics.

In this study, the EMFs showed a strong enrichment of the ID markers n-cadherin and Cx43 (Figs 1B, 1D, 2 and 3), compared to the CF, thereby confirming our followed procedure. The presence of a large number of cytoplasmic, cytoskeletal, mitochondrial and endoplasmic reticulum proteins, however, also revealed that the EMF fraction was constituted by a variety of membrane proteins. Therefore we relied on an intuitive stringent filtering approach, which was based on the enrichment ratio, consistency between the three replicates and specific documented features of subcellular localization and functions. In this way we were able to isolate a strong set of known ID proteins in our dataset (Fig 2, e.g. Cx43, n-cadherin) along with several promising candidates (Fig 3) that were also localized to the membrane. Of note, this stringency in selection may have resulted to the exclusion of other putative candidates, but also of already confirmed ID-proteins like plakophilin. Although we detected plakophilin in our EMF fraction with an ER = 34, it was not very abundant (not many peptides) and only detected in one ID fraction and one CF fraction, thereby not making it to the final stringent candidate list.

In this final list of 97 potential candidates, 27% of the candidates were documented junction proteins further strengthening the basis of our filtering approach and increasing the potential of the new candidates to be actual ID proteins. Based on ER values signifying enrichment we selected Flotillin-2 (ER = 100) as a potential ID protein. Flotillin-2 is a member of the flotillin family, which is generally known to function as a caveolin functional homolog [35]. It is abundantly expressed in striated muscle tissue and has been detected in extracts from both mouse myocardium and neonatal cardiomyocyte structures [36]. We were able to confirm the presence of flotillin-2 in the ID via immunohistology (Fig 5 and S1C Fig). Flotillin-2 presented exclusive localization at the cell junctions of human and rat cardiomyocytes and was heavily enriched in our proteomics data from rat LV tissue. As of now, not much is known about the function of this protein at this location in the myocardium. Therefore, to further highlight its putative importance we investigated its behavior in tissue affected by cardiac disease such as DCM and ARVC, since under such pathological conditions, alterations in composition of the ID play an important role in manifestation of the clinical characteristics. These studies clearly showed increased levels of flotillin-2 at the ID in these diseased hearts.

We have proposed another heavily enriched candidate nexilin (ER = 68) to be a member of the ID. Nexilin is a known cardiac Z-disk protein and mutations in this protein have been linked to dilated cardiomyopathy by destabilizing the Z-disk [37]. The ID has been suggested to be similar to a giant Z-disk [22], and hence it becomes quite reasonable to assume that these structures may have similarities in protein composition. Also, at the intracellular end of the ID, the sarcomere interfaces the ID with a final sarcomeric Z-disk.

POPDC2, popeye domain containing protein 2, and its close homologue POPDC1 (also present in our data) are abundantly expressed in the heart and in cardiomyocytes [38]. Both are sarcolemmal proteins with the so-called popeye domain pointing inwards. This domain was recently established as a high-affinity binder of the signal molecule cAMP. POPDC1 is a known cell-cell contact protein in epithelial cells. Recent work has established POPDC1 and POPDC2 as important mediators of cardiac pacemaker function by interacting with potassium channels, and functionally manipulating these currents. POPDC2-/- mice suffer from age-dependent bradyarrhythmia starting at 8 months of age [38]. A current role for POPDC2 in myocytes of the working myocardium has not been established yet. Here we now implicate both popeye proteins as members of the ID, where it is tempting to speculate a functional role in conduction and cAMP signal transduction. Very recently, for POPDC1 and POPDC2 it was shown that these proteins could be visualized in the intercalated disk, while POPDC2 was also present at the lateral membranes of ventricular cardiomyocytes [39,40].

For the thioredoxin-related transmembrane protein 2 (TMX2), it is currently unclear what its function is. Based on similarity, Uniprot database suggests no thioredoxin activity for this particular membrane embedded protein. Therefore it may have a different function. In general, TMX2 expression is ubiquitous, but in heart and cardiomyocytes it clearly enriches at the cell-cell junctions.

Not all of the four candidates showed exclusive ID staining as some also revealed some lateral membrane localization of minor intensity, however not in all species studied (e.g. POPDC2 (Fig 4) and NEXN (S1 Fig)). The differences in signals as observed in the three species might reflect true differences in subcellular localization of the proteins. On the other hand, these differences might also have been influenced by technical issues. It is for example well known that antibody-epitope interactions may vary between species due to minor sequence variations. This can affect the signal-to-noise-ratio. Nonetheless, all four candidates stain the ID region most strongly and according to the quantitation overlap (S2 Fig), strong correlations with N-Cadherin were observed.

It is currently not feasible to fully enrich the ID while at the same time not enriching for other membrane compartments. The above-presented results not only highlight the difficulties in studying the ID composition but also show how the use of advanced proteomic tools and bioinformatics allow us to get closer to deciphering the composition of the ID. A limitation is that we were only able to pinpoint membrane embedded proteins, while remaining blind to ID associated proteins. In addition we can not exclude that by filtering, we have eliminated proteins from our prioritized list from which location/function has been allocated to intracellular membrane compartments without knowledge of a potential role at the ID. Also filtering settings needed to be stringent to keep false positive levels low. This being said, it is very encouraging to see that all 4 candidates investigated further in this study proved indeed to localize in the ID along with known markers. Therefore this work provides the first, experimentally based compendium of 97 (putative) ID proteins.

## Supporting Information

S1 Fig Co-localization of candidate proteins with n-cadherin. (A) Co-localization of N-cadherin (left) with TMX2 (middle) in the ID of human left ventricular tissue, rat left ventricular tissue and isolated dog cardiomyocytes. (B) Co-localization of NEXN and N-cadherin in rat left ventricular tissue and isolated dog cardiomyocytes. (C) Immunofluorescence data confirms co-localization of flotillin-2 with N-cadherin in the ID in other species (rat left ventricular tissue and isolated dog cardiomyocytes.
(TIFF)

S2 Fig Quantification of Co-localization in DCM patients. Comparison of the N-cadherin and flotillin-2 fluorescence profiles with ImageJ. A line was plotted through the visible fluorescence signals to create a plot profile of signal intensities along the line. The profiles were compared and correlated (R^2^).
(TIF)

S3 Fig Original Western blots. Orginal uncropped Western blots that were used to generate the Figs 1B (I), 4A (II) and 5C (III).
(TIFF)

S1 Table A: Protein identifications in EMF and CF. Protein identifications in EMF (EMF1-3) and CF (C1-3) based on the MaxQuant output. Depicted is the label-free quantification data. Highlighted in yellow columns are the criteria used for candidate selection. B: ID candidate proteins and cellular localization. MaxQuant data of the 366 ID candidate proteins evaluated for cellular localization as displayed in Fig 2. C: Final list of ID candidate proteins. 97 candidate ID proteins including their enrichment ratio (ER), presence in the Estigoy et al. paper [22], and annotation as junction protein in Gene Ontology terms.
(XLS)
