# Supplementary figures and images for: A Proteomics Approach to Identify New Putative Cardiac Intercalated Disk Proteins
Source: PLoS One. 2016 May 5;11(5):e0152231. doi: 10.1371/journal.pone.0152231 (PMC4858182; doi:10.1371/journal.pone.0152231)

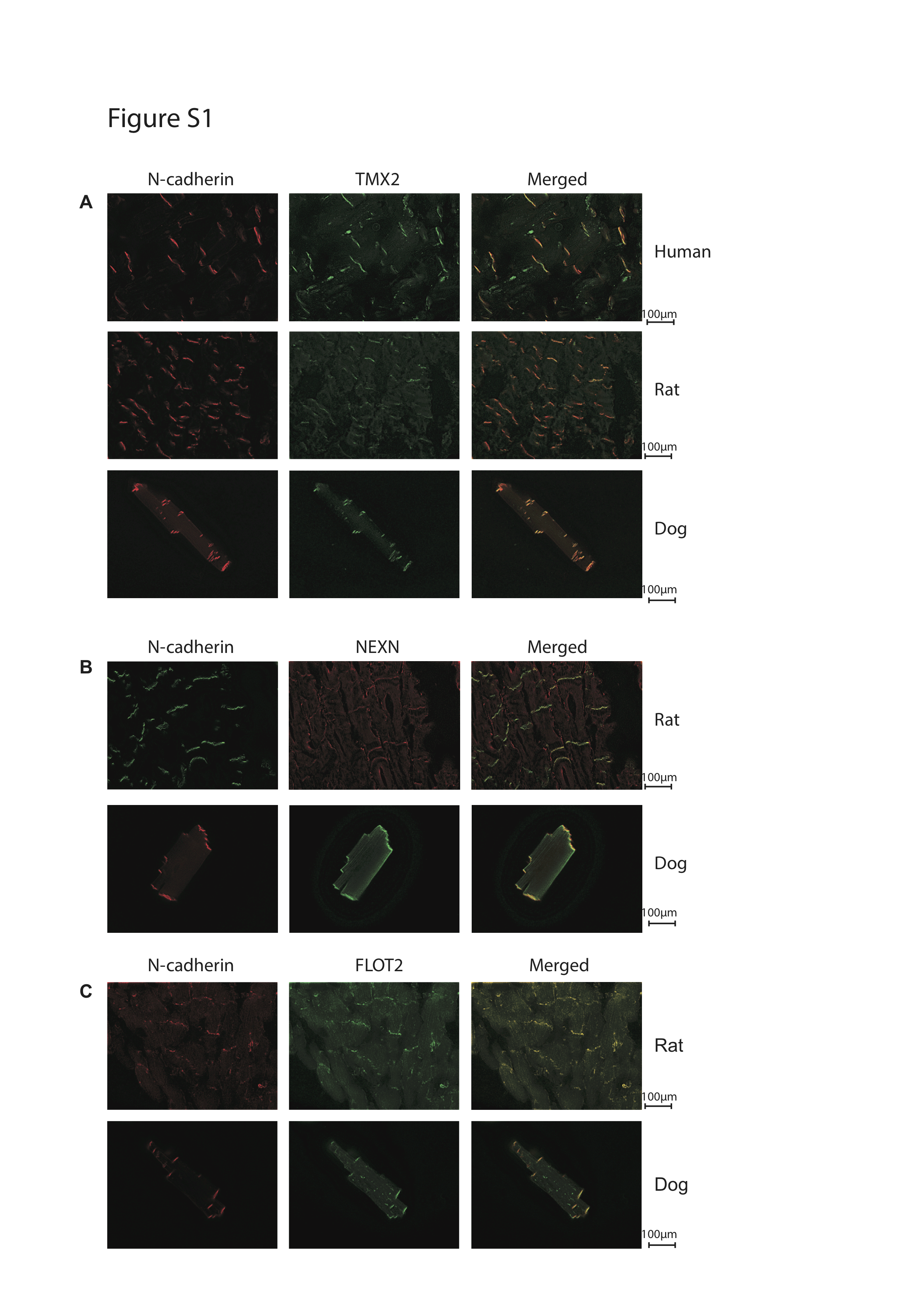

Supplement: S1 Fig — (A) Co-localization of N-cadherin (left) with TMX2 (middle) in the ID of human left ventricular tissue, rat left ventricular tissue and isolated dog cardiomyocytes. (B) Co-localization of NEXN and N-cadherin in rat left ventricular tissue and isolated dog cardiomyocytes. (C) Immunofluorescence data confirms co-localization of flotillin-2 with N-cadherin in the ID in other species (rat left ventricular tissue and isolated dog cardiomyocytes. (TIFF) [file pone.0152231.s001.tiff]

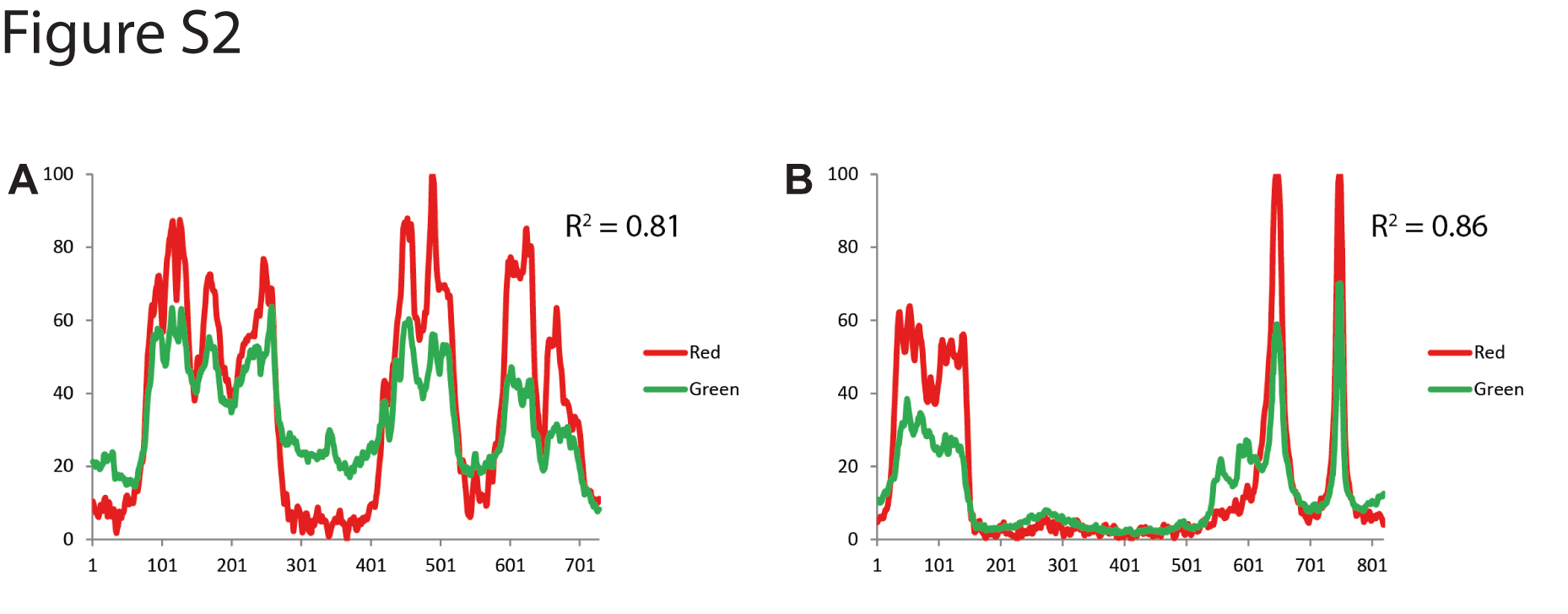

Supplement: S2 Fig — Comparison of the N-cadherin and flotillin-2 fluorescence profiles with ImageJ. A line was plotted through the visible fluorescence signals to create a plot profile of signal intensities along the line. The profiles were compared and correlated (R2). (TIF) [file pone.0152231.s002.tif]

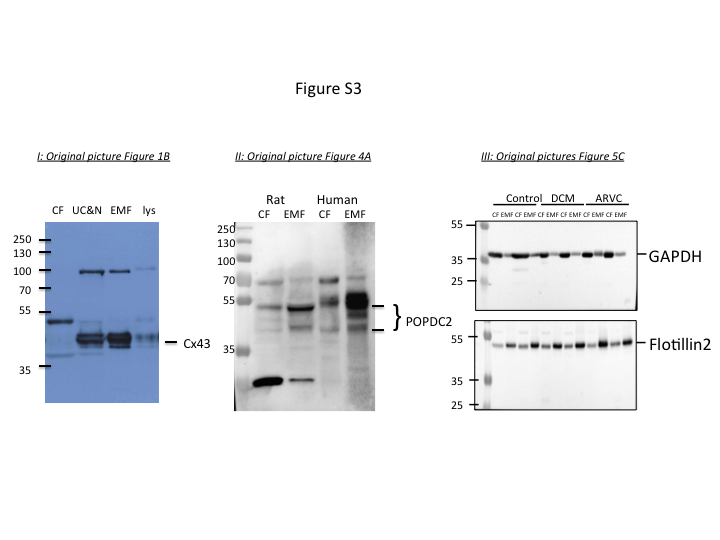

Supplement: S3 Fig — Orginal uncropped Western blots that were used to generate the Figs 1B (I), 4A (II) and 5C (III). (TIFF) [file pone.0152231.s003.tiff]
